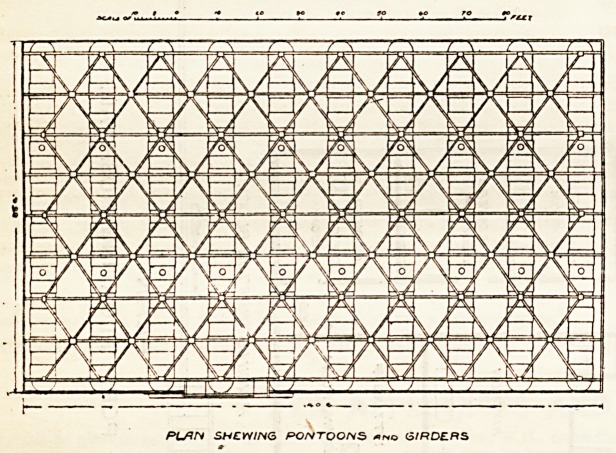# Hospital Construction

**Published:** 1896-01-11

**Authors:** 


### The Institutional Workshop.

### HOSPITAL CONSTRUCTION.

### THE FLOATING HOSPITAL ON THE RIVER TEES.

UNTIL the year 1892 the only provision made by the River Tees Port Sanitary Authority in the shape of hospital accommodation was the hull of an old sailing vessel, moored in the river near Newport and fitted up for six beds; in the autumn of that year, repairs having become urgent, the need for a suitable hospital was strongly represented to the authority by their medical officer, Dr. Malcolmson, and in consequence Mr. W. G. Laws, the city engineer of Newcastle-on-Tyne, was called in to give advice. His recommendation to build a floating hospital has been duly carried out from excellent plans prepared by Mr. Stainthorpe, engineer and surveyor to the Urban

District Council, the contract being given to Messrs. Head, Wrightson, and Co., of Thornaby-on-Tees.

The site selected, about half-a-mile below the Eston Jetty, was obtained by arrangement with the Tees Conservancy Commissioners. Here, inside a crescent-shaped breakwater, constructed of slag, as a protection from north and north-east gales, is anchored the new hospital. The platform or "float" upon which the buildings have been erected measures about 140 ft. by 86 ft., and is formed of wooden planks, three inches thick, resting on cross-braced girders and wood joists attached to, and buoyed up by, ten rivetted iron pontoons, each 86 ft. 6 in. by 6 ft. diameter. The height of the deck above the water level is 4 ft., and a sloping gangway leads to a broad landing which is level with the deck of the steam launch belonging to the authority which conveys patients from the ships to the hospital, so that easy transference of the sick from the one to the other is secured.

The buildings erected on the float consist of two ward blocks and an administrative block midway between them, behind which are laundry, mortuary, and destructor. The ward blocks are each 68 ft. long

PLAN SHEWING PONTOONS AND GIRDERS

by 24 ft. 6 in. wide. No. 1 block contains one large ward for eight beds and one small ward for two beds, this latter to be used as a receiving or isolation room, where patients will be kept until their cases can be properly diagnosed. Both wards are provided with w.c. and scullery in small blocks attached to the main building. Between these wards are the nurses' room and bath-room. A covered verandah gives access to the entrance lobbies of the two wards, and also to the nurses' room. No. 2 block has also two wards, one for four and one for six beds, the arrangement of nurses' room, bath-room, &c., being identical with block No. 1. The administrative block is 58 ft. by 30 ft., and is divided by an open space of 28 ft. on each side from the ward blocks. In it are the matron's sitting-room (which will be used also as the dining-room), kitchen, scullery, larder, stores, &c., a room for the medical officer, and four double-bedded rooms for the matron, day and night nurses, and servants. The medical officer's room is so arranged that he need not pass beyond the front lobby to gain access to it.

Generally, we may state that the situation of the w.c.'s, especially in the administration block, leaves something to desire; the bedrooms for the staff are too small, and the baths are so placed in the ward bathrooms as to prevent access to one side, which is barred by the lavatory basins.

The laundry has been fitted with all the latest appliances by Messrs. Bradford and Co. The mortuary is a small detached building, with flagged floor, and lighted by open skylights. The destructor is a specially-constructed furnace, built in fire-brick, and provided with suitable fire-bars for burning infected bedding, refuse, &c., supplied by Messrs. Goddard, Massey, and Warner, of Nottingham.

The wards are heated by means of "Musgrave's Ulster Stoves," one in each of the small wards and two in the larger ones. They are covered with glazed tiles, and are provided with a locking arrangement, of which the nurse keeps the key, as a precaution against anyone playing or interfering with the fires.

Great care has been taken to secure thorough ventilation in the wards. The upper portions of the windows have a swing sash opening inwards, worked by Leggatt's fan-light opener. Under the head of each bed, and level with the floor, is fixed one of Kite's fresh air inlet ventilators. Vitiated air is extracted by ventilating shafts, 18 in. square, carried through the roof, and widening out to 3 ft. square at the ceiling level, while through these shafts the flues from the stove pipes are fixed, aiding the ventilation by an upward current. The floors of each block are raised 9 in. above the deck level, so that there is ample space for a current of air beneath.

A 300-gallon cistern is placed in the roof of each block for fresh water supply, the fresh water being brought down the river in a proper water boat and pumped into the cistern. Hot and cold water is freely laid on in bath-rooms and sculleries, and the wards are provided with portable baths on rubber-tyred wheels. River water supplies the cisterns for the w.c.'s and slop hoppers, while rain water from the roof is collected and pumped to a cistern over the laundry for use therein.

The lighting of the hospital has to be done by oil ships' lamps, hung on pivots, as it is impossible to obtain a gas supply. A small lamp-room, properly fitted up, is at the rear of the administration block, but it should have been placed apart from any of the main buildings.

The buildings are lined throughout with match boarding. Some more serviceable finish for hospital work might, we should think, have been found than this, and more worthy of the plans we publish herewith.

The total cost of the hospital and the works in connection therewith has been approximately £10,000. The wards will be fitted up with the latest improvements in beds and furniture at an estimated cost of about £200.

## Figures and Tables

**Figure f1:**
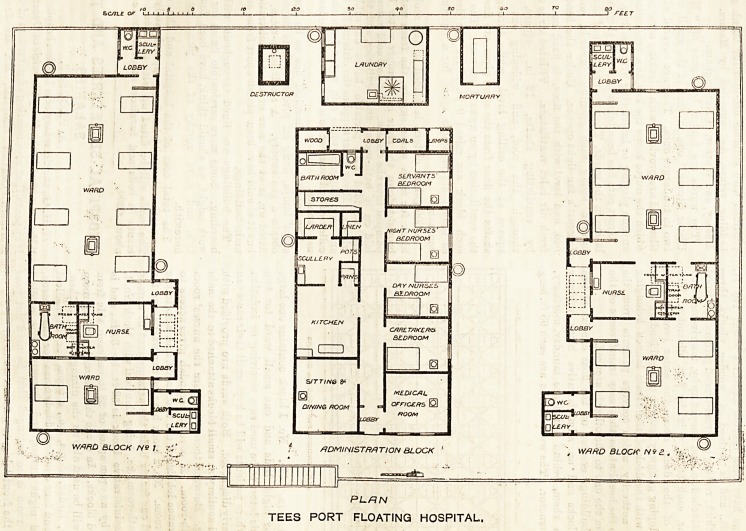


**Figure f2:**